# Mechanisms of miRNA-Mediated Gene Regulation from Common Downregulation to mRNA-Specific Upregulation

**DOI:** 10.1155/2014/970607

**Published:** 2014-08-10

**Authors:** Ayla Valinezhad Orang, Reza Safaralizadeh, Mina Kazemzadeh-Bavili

**Affiliations:** Department of Animal Biology, Faculty of Natural Sciences, The University of Tabriz, Tabriz, Iran

## Abstract

Discovered in 1993, micoRNAs (miRNAs) are now recognized as one of the major regulatory gene families in eukaryotes. To date, 24521 microRNAs have been discovered and there are certainly more to come. It was primarily acknowledged that miRNAs result in gene expression repression at both the level of mRNA stability by conducting mRNA degradation and the level of translation (at initiation and after initiation) by inhibiting protein translation or degrading the polypeptides through binding complementarily to 3′UTR of the target mRNAs. Nevertheless, some studies revealed that miRNAs have the capability of activating gene expression directly or indirectly in respond to different cell types and conditions and in the presence of distinct cofactors. This reversibility in their posttranslational gene regulatory natures enables the bearing cells to rapidly response to different cell conditions and consequently block unnecessary energy wastage or maintain the cell state. This paper provides an overview of the current understandings of the miRNA characteristics including their genes and biogenesis, as well as their mediated downregulation. We also review up-to-date knowledge of miRNA-mediated gene upregulation through highlighting some notable examples and discuss the emerging concepts of their associations with other posttranscriptional gene regulation processes.

## 1. Introduction

MicroRNAs or miRNAs have been a subject of significant research work since the discovery of lin-4 in the early 1990s, underscoring the importance of posttranscriptional gene regulation in cis and trans [1]. miRNAs are a subset of endogenously-initiated, single-stranded noncoding RNA guide molecules, traceable in organisms as diverse as animals, plants, green algae, and viruses, that regulate gene expression via association with effector complexes (called “micro-ribonucleoprotein” or “miRNP”) and sequence-specific recognition of target sites (also called “cognate mRNAs”), which can dictate the functional outcome [2–5]. They represent one of the most exciting areas of modern medical sciences as they possess unique ability to modulate an immense and complex regulatory network of gene expression [6, 7] in a broad spectrum of developmental and cellular processes including tissue development [8, 9], cell proliferation [10, 11], cell division [12, 13], cell differentiation [14], neuronal asymmetry [15], metabolism [16], stem cell properties [17], apoptosis [18], protein secretion [19], and viral infection [20]. It is becoming clear that they have a big impact on shaping transcriptomes and proteomes of eukaryotes [21]. Aberration or perturbation in their expression levels has significant correlation with serious clinical consequences, including disease of divergent origin and malignancy [22, 23]. Certainly, disease-associated miRNAs represent a substantial class of targets for the miRNA-based novel therapeutic or diagnostic/prognostic biomarkers [24, 25]. By mid-2013, it was known that the human genome encodes over 2000 different miRNAs that scattered on all human chromosomes except the Y chromosome (http://microrna.sanger.ac.uk; Release 20: June 2013). Based on this estimation, about 3-4% of human genes encode miRNA. In postgenomic era, the accepted notion is that a single miRNA species can regulate hundreds of targets, even if only to a mild degree, but, conversely, several miRNAs can bind to their target mRNAs and cooperatively provide fine-tuning of a single mRNA target expression [26]. Although a steeply growing computational analysis has identified a range of potential targets for miRNAs, to date, only a small number of them have been validated by experimental approaches [27, 28].

Until recently, miRNAs had been believed to have a pervasive effect on the gene expression modulation solely by negative regulation of target mRNA [29]; however, the increasing published observations indicate that miRNAs oscillate between repression and stimulation in response to specific cellular conditions, sequences, and cofactors [30]. These exciting findings, however, have made it even more difficult to explain how miRNAs regulate gene expression. While the past decades have witnessed a veritable exploration focuses on defining the regulatory function of miRNAs, fewer directed towards exact mechanistic turnovers under specific cellular conditions and many of these assertions directly contradict one or another of the publications. Hence, undoubtedly, there are still enigmas to be uncovered regarding mechanistic details of miRNA-mediated regulation.

In order to exploit practical implications of miRNAs as biomarkers, novel drug targets, and therapeutic tools for diagnosis, prognosis, and treatment of malignancies and disease, it is necessary to have in-depth understanding of miRNA turnover, in particular, the molecular mechanisms by which miRNAs elicit distinct gene expression outcomes in different cell cycle stages and conditions. Toward this end, our review illuminate and explain the controversies generated by recent assertions as well as providing a comparison of regulatory switches that mediate between downregulation and upregulation, directed by miRNAs. This review, therefore, aims to summarize new findings in miRNA-mediated gene regulation mechanisms, which may be affected by different cellular conditions and specific transcripts and proteins.

## 2. miRNA Genes and Biogenesis

Central to studying miRNA-mediated gene modulation is the clear understanding of their gene structure and biogenesis, which have been described in several reviews [31–33]. miRNA genes are distributed nonrandomly in human genome, and nearly half of them are found as tandem arrays within clusters, sharing the same promoter, which may indicate gene duplications [15, 34]. It has been shown that miRNA genes frequently coincide with tumor susceptibility loci [35] and are located within known sites of retroviral integration [36] and deleted or amplified genomic region or break points [37], as well as inside or near homeobox (HOX) cluster [38].

By combining up-to-date genome assemblies and expressed sequence tag (EST) database, it was proved that miRNA promoters can be divided into two broad categories: intergenic and intragenic [39, 40]. Intergenic miRNAs are located between genes and their transcriptions are independent of coding genes since they are transcribed mostly by RNApol III. Therefore, they have been reported to be more evolutionary conserved [41, 42]. However, intragenic miRNAs are embedded within exon or introns of protein-coding genes, and thus, are coexpressed in the same orientation of their host genes by RNApol II [43]. Additionally, a small percentage of miRNAs are found interspersed among repetitive elements that are transcribed by RNApol III and, subsequently, processed in the same way [44].

Experimental evidence revealed that sequential processing of miRNAs occurs first in the nucleus and then in the cytoplasm. The biogenesis of miRNAs starts with RNApol II or III dependent transcription of a miRNA gene locus generating a long primary RNA (pri-miRNA). Pri-miRNAs are 5′-7-methylguanosine capped, spliced and 3′-polyadenylated and have a coding capacity for one or more mature miRNAs [2, 4, 45]. They can reach several kilobases in length [46]. Pri-miRNAs are processed into smaller, hairpin-like miRNA precursors called pre-miRNAs [47, 48]. The cleavage of pri-miRNAs proceeds contemporaneously with the transcription of the genes or ncRNAs [49]. After nuclear processing, Pre-miRNAs are transported by Exportin-5 to the cytoplasm in a Ran guanosine triphosphate-dependent manner, recognizing characteristic hairpin stem and 3′ protruding overhang in the pre-miRNA [50, 51]. Further cytoplasmic processing performs a second cleavage of the hairpin structure and defines the 5′ end of the mature miRNAs. This releases a 19–24 nucleotide double-stranded RNA where miRNA is the antisense or guide strand and miRNA∗ is the sense or passage strand [52]. Once cleavage is occurred double-stranded miRNA (dsmiRNA) is released and integrated into the appropriate effector complex. Afterward, the duplex is unwound and the miRNA∗ strand becomes degraded leaving one fully mature miRNA strand and finally microRNA containing ribonucleoprotein (miRNP) is configured [53, 54].

## 3. miRNA-Mediated Downregulation

miRNAs are indeed able to reduce gene expression by multiple pathways and modes [55]. Striking observations suggested that miRNAs do not function as naked RNAs, instead, they function in the form of effector complexes, which are known as miRNP, miRgonaute, or miRISC, along with Argonaute, the most important constituent of all miRNPs [56]. A key specificity determinant for miRNA target recognition is based on Watson-Crick pairing of 5′-proximal “seed” region (nucleotide 2 to 8) in the miRNA to the seed match site in the target mRNA, which positioned mostly in 3′UTR [57]. Nevertheless, it is also claimed that a small subset of miRNAs modulate expression by specifically targeting the 5′UTR and/or coding region of some mRNAs [58, 59].

**Figure 1:**
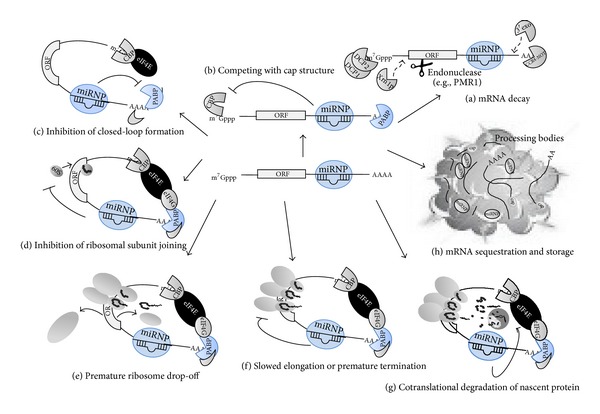
Potential mechanisms of miRNA-mediated downregulation. Ribonucleoproteins (miRNPs) target mRNAs through sequence complementation. The association between miRNPs and mRNAs can have one or several consequences. miRNP-mediated downregulation may have direct and indirect mechanisms and occur before translation is triggered or after translation initiation. (a–c) Translation initiation mechanisms. miRNP interferes with early steps of translation before elongation. (a) miRNPs recruit several factors and enzymes for mRNA cleavage and degradation including decapping enzymes, deadenylase, 3′ and 5′ exonucleases, and endonucleases. (b, c) Argonaute protein competes with cap binding proteins (CBPs) and elf4E for binding to cap structure and inhibits translation initiation by interfering with mRNA circularization and formation of closed-loop achieved through cap structure interaction with CBPs and elf4E/G required for translation initiation. (d–h) Postinitiation mechanisms. miRNPs repress translation elongation and termination or involve in protein degradation and sequestration. (d, e) miRNP interferes with ribosome subunit by inhibiting its joining or promoting its dissociation. (f, g) miRNP obstructs translation elongation by competing with elongation factors or cotranslationally recruiting protein decay factors such as exosomes. (h) Target mRNA is sequestered from translation machinery and stored or sometimes is degraded by enzymes. However, alternatively, translationally inhibited mRNA along with associated proteins could be sequestered at the same bodies. For explanations in support of illustrations, refer to the main text.

The biological outcome of miRNA-mRNA interaction can be altered by several factors contributing to the binding strength and repressive effect of a potential target site [60]. The most mentioned factor is perfect base pairing between the miRNA seed region and target site [61]. Other factors include the number of target sites for the same miRNA and relative position of them, site accessibility, sequences flanking miRNA target site, and their context, and RNA secondary structure can influence the consequences of hybridization [62–64]. The interaction between the effector complex carrying miRNA and target mRNA can have several consequences. To date, several and in some cases contrary models have been proposed, although they are not mutually exclusive. Briefly gene silencing mediated by miRNA can be obtained at three stages that include pretranslational, cotranslational, and posttranslational steps and can exert direct and indirect effects on translation machinery (Figure 1).

In the case of pretranslational step, a number of reports suggest that in certain organisms, such as mammalians, a specialized RNA-induced transcriptional silencing (RITS), which contain specialized nuclear Argonaute (Ago) protein, may results in gene silencing through chromatin remodeling [65, 66].

Beside transcriptional effects, miRNAs can repress translation initiation by multiple mechanisms. mRNA degradation can be inaugurated by deadenylation from 3′ end and/or decapping from 5′ end by enzymes such as DCP1/2. Missing poly(A) tail and cap structure expose the remained RNA for the action of degradation of exonucleolytic Xrn1p enzyme. In addition, truncated mRNA, missing poly(A), can be subjected to the 3′ to 5′ degradation of cytoplasmic exonucleases [67–69]. Alternatively, sequence-specific endonucleolytic mRNA cleavage by polysomal ribonuclease 1 (PMR1) may occur in parallel [70] (Figure 1(a)). Recruited Argonaute protein interacts with several initiation factors [71]. First and foremost, Ago competes with eIF4E, an eukaryotic translation initiation factor involved in directing ribosomes to the cap structure of mRNAs, for binding to cap structure [72, 73] (Figure 1(b)). Other translation initiation factors include: PABP (the protein associated with poly(A) tail at 3′-end of mRNA), eIF4G which is strongly associated with eIF4E, the RNA helicase that unwinds mRNA secondary structure, eIF4A which is required for the binding of mRNA to 40S ribosomal subunits, and eIF3 and eIFa which are associated with the ribosomal small subunits [74]. It has been also reported that translation inhibition could happen when target sites for miRNA are located in 5′UTR or even coding sequences [75].

Another possible mechanism of translation initiation blocking is the Ago interference with the formation of closed-loop mRNA, achieved through interaction between cytoplasmic poly(A) binding protein and cytoplasmic cap binding protein, by an ill-defined mechanism that may include deadenylation [73] (Figure 1(c)).

In postinitiation steps, eIF6 is recruited by Argonaute, which prevents the large ribosomal subunit from joining to miRNA-targeted mRNA [76, 77] (Figure 1(d)). In addition, miRNPs interfere with elongation factors and lead to ribosome subunit dissociation and/or premature termination [71] (Figures 1(e) and 1(f)). Along with translation repression, cotranslational protein degradation may occur. In this model, the nascent polypeptide is degraded by protease activities [78] (Figure 1(g)).

Recently, it was indicated that processing cytoplasmic foci, mostly known as “P or GW bodies,” have a central role in mRNA degradation and translation inhibition. Target mRNAs associated with P body components can either be degraded or be sequestered to return to translation. Therefore, the rates of expression and degradation of mRNAs are influenced by a dynamic equilibrium between polysomes and miRNPs that are found in P bodies. Moreover, some mRNA-specific regulatory factors, including miRNAs, appear to repress translation and promote decay by recruiting P body components to specific mRNAs since they contain enzymes involved in mRNA turnover, such as Argonaute and GW182. Collectively, these findings have been converged to propose a possible model in which targeted mRNA is sequestered from the translational machinery and underwent both degradations or is stored for subsequent processes [79–81] (Figure 1(h)).

## 4. miRNA-Mediated Upregulation

In contrast to general assumption that miRNA-mediated downregulation is a one-way process and leads to decreased mRNA stability and/or translational inhibition, Vasudevan and Steitz reported for the first time that the miRNA-mediated downregulation is reversible [82]. Likewise, there is evidence suggesting that some miRNAs could upregulate gene expression in specific cell types and conditions with distinct transcripts and proteins.

In miRNA-mediated upregulation, miRNPs act in trans in promoting their target mRNAs' expression similar to miRNA-mediated downregulation. The mRNA expression could be activated by the direct action of miRNPs and/or could be indirectly relieved from miRNA-mediated repression by abrogating the action of repressive miRNPs [82].

In addition, a single miRNA can act both in up- and downregulation, and likewise a single specific gene could encounter both regulation directions based on the specific conditions and factors. For instance, miR-145 mediates myocardin gene upregulation during muscle differentiation. However, ROCK1 expression downregulation is a consequence of miR-145 targeting in osteosarcoma [83, 84]. As another example, KLF-4 is upregulated by miR-206 in confluent and nontumor cells, while it is downregulated by miR-344 in proliferating and normal cells [85]. Therefore, these evidences confirm that gene expression upregulation is specific to cell type, cell condition, and present factors and elements.

### 4.1. Direct Mechanisms of miRNA-Mediated Upregulation

Complexity of gene regulation by miRNAs is further expanded by observations that miRNAs can positively mediate gene expression. These reports indicate that posttranscriptional upregulation by microRNAs is selective, specific to the RNA sequence context, and associated with miRNP factors and other RNA binding proteins [86]. Similar to miRNA-mediated downregulation, translation upregulation by miRNAs has been observed to range from fine-tuning effects to significant alterations in gene expression levels. These studies uncover remarkable capability of some miRNAs and their associated miRNPs in gene expression control and highlight the importance of regulation in directing appropriate microRNP responses [87].

#### 4.1.1. miRNA-Mediated Upregulation in Response to Cellular State and/or in the Presence of Specific Factors

The miRNP and target mRNA base pairing could have several and in some cases converse functional outcomes. Several studies provided evidence that miRNPs have the potential of activating gene expression in the presence and/or absence of specific factors and through distinct cell conditions. They revealed that cell cycle has the potential to determine miRNA-mediated gene regulation direction by promoting or inhibiting special mRNA expressions.

One of the deeply studied factors is the effect of G0 state on miRNA-mediated gene regulation [30]. Quiescence generally refers to G0 and G0-like states that run a specific gene expression programs in order to enter the G0 cells for extended period of time in a reversible manner. G0 state can be observed during natural phenomena such as differentiation, development, and growth to confluence or can be induced by the manipulation of in vitro cell culturing [88–91].

Translational activation in substitute cell states, for instance, G0 and immature oocyte, provides a mean of gene expression to skew towards maintaining the state. Loss of these states leads to the reversion of miRNA-mediated gene expression activation [92].

In drosophila, it was proved that both AGO1 and AGO2 are capable of mediating gene expression downregulation. Nevertheless, AGO2, but not AGO1, can be involved in gene expression activation when their targeted mRNA lacks poly(A) tail, representing the significance of the lenghth of poly(A) tail for the diverse roles of AGO2 [93, 94].

What is more, AGO2-RISC binds to eIF4E and is capable of forming “closed-loop” even in the absence of poly(A) tail and associated proteins and, hence, could activate translation directly [72]. Furthermore, GW182, a crucial protein for miRNA-mediated downregulation, was reported to be downregulated in G0 state and immature oocyte. As a result, GW182 misses its interaction with AGO and leaves the capability for another protein named Fragile-X mental retardation protein 1 (FXR1) to be involved in miRNP complex. This eventually results in miRNP-mediated gene activation [95]. Furthermore, biochemical experiments revealed that AGO2 is too small to contain GW182, whereas AGO2-FXR1-iso-a is a complex naturally found in nuclei. Therefore, lacking GW182 results in abrogation of expression downregulation, while FXR1 association with AGO2 leads to translational activation [82, 96].

Nuclear events are often dictated the fate of mRNA expression; in line with this, miRNPs' responses and remodeling for different cell states can comprise a nuclear phase for G0 [97, 98]. Slicer activity of Argonaute protein has been reported to be absent in immature oocyte and G0 state [89, 99]. Thus, immature oocyte and G0 state cells recruit miRNAs to conduct their cleavage-independent regulatory roles (e.g., translational activation).

**Figure 2:**
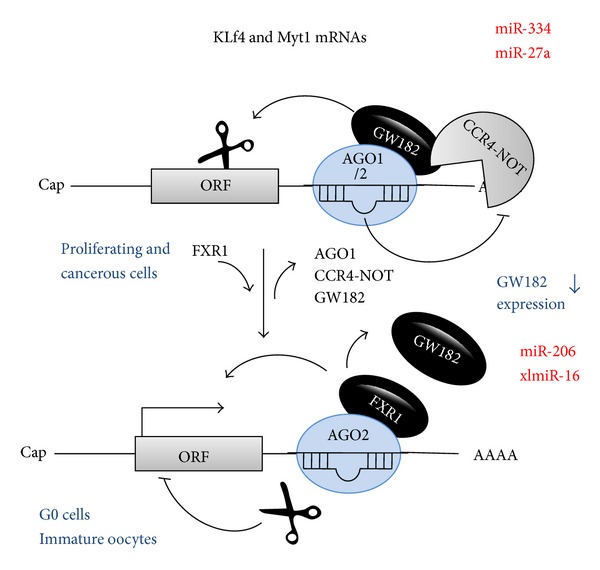
miRNA-mediated upregulation according to cell cycle phase. miR-334 and miR-27a bind to the 3′UTR of KLF4 and Myt1 mRNA in proliferating and cancerous cells and subsequently downregulate mRNA expression by both translational inhibition and mRNA degradation. miRNP-mediated repression requires factors including AGO, GW182, deadenylases, and nucleases. However, in G0 cells and immature oocytes, GW182 is downregulated and FXR1 is associated with AGO2 bearing miRNP and leads to gene expression upregulation. miR-206 is associated with KLF4 mRNA in G0 cells and xmiR-16 is associated with Myt1 mRNA in immature* Xenopus oocyte*. miR-206 and xmiR-16 activate KLF4 and Myt1 expression, respectively, by binding to their 3′UTRs and recruiting special factors such as FXR1, since AGO2 is associated with gene expression activation in these cells and lacks slicer activity.

Both cells at G0 state and immature oocytes have an intact nucleus [100, 101]. Elevated numbers of activator miRNPs and FXR1-iso-a are naturally compartmentalized in the nucleus, which then act as selective activators of target mRNAs [102]. Examples are KLF4 mRNA and miR-206 in quiescent cells and Myt1 and miR-16 in immature oocytes, which are illustrated in Figure 2 [85, 100, 103, 104].

**Figure 3:**
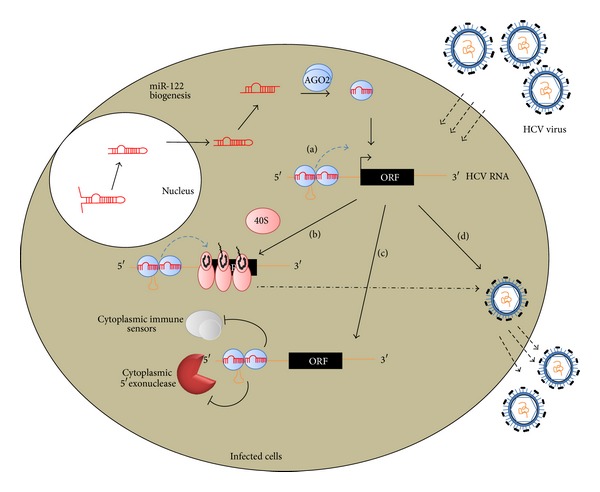
Activation of hepatitis C virus (HCV) expression by miR-122, a liver-specific microRNA. The mature single-stranded miR-122 is processed from its double- stranded precursor and incorporated into functional miRNP, bearing AGO2. miRNPs target one or two tandem binding sites in the 5′ noncoding region (5′NCR) of HCV RNA. HCV appears to usurp the miRNP to increase its RNA accumulation by a number of mechanisms. (a) First, miR-122 complexes provide a scaffold for the binding of essential factors such polymerase for RNA replication. (b) In addition, miRNP complexes increase the association of 40s ribosome subunit, and thus, result in increased translation and protein yield. (c) They also form an unusual oligomeric complex in 5′ end of HCV RNA and result in HCV RNA stability enhancement by masking its single-stranded 5′end through hiding 5′NCR from 5′ cytoplasmic exonucleases and immune sensors. (d) Also, miR-122 binding was reported to lead to increased propagation and life cycle of virus by some ill-defined mechanisms.

miRNAs usually interact with 3′UTR of target mRNAs leading to mRNA degradation and/or translational repression. In contrast, it was recently exposed that liver-specific miR-122 enhances hepatitis C virus (HCV) RNA levels via interacting with two natural binding sites in 5′ noncoding region of RNA [105]. miR-122 expression was found to be specific to liver cells. It was reported that increasing levels of miR-122 are in accordance with differentiation of liver tissues in developing mouse embryos and contribute pointedly to HCV liver tropism [106]. These tandem miRNA binding sites are located in the upstream of the internal ribosome entry sites (IRESs), where miRNA-binding interference is eliminated and translation gets the chance to initiate. In line with this, miR-122 interaction with a manipulated binding site in HCV RNA which is located in 3′UTR of a reported gene revealed an expression downregulation, indicating the location specificity of miR-122-mediated upregulation in HCV gene [105, 107]. The mechanism of the miR-122-mediated stimulation of HCV gene expression is one of the rare cases and remains partially unsolved [108]. However, the emerging experiments and mutational analysis validated that both miR-122 seed sequence binding and extra interactions are needed to act cooperatively in order to enhance viral RNA abundance [109]. Since HCV genome, as a viral genome, does not have cap structure and thus misses the associated proteins at its 5′ end, it eventually requires alternative mechanism to promote translation by recruiting translational components, eventually leading to increased RNA stability by inhibiting exonucleases digestion [69]. Indeed, it is proposed that miR-122 acts instead of cap structure in enhancing RNA expression by increasing its stability against Xrn1, accelerates the binding of ribosome, and exerts another Xrn1-independent role in stimulating HCV gene expression [110, 111]. Components such as RISC which are brought to HCV genome by miRNP would function as a shield in protecting single-stranded 5′ end of HCV from cytosolic exonucleases activities [111]. In addition, these proteins may provide a scaffold for binding of factors essential for RNA replication and translation [112].  Figure 3 represents the different mechanisms of miR-122-mediated HCV gene upregulation.

Another outstanding miRNA-mediated upregulation would be related to miRNAs targeting TOP mRNAs. TOP mRNAs commonly foster a 5′ terminal oligopyrimidine tract (5′TOP), which is a structural trademark comprising the core of the cis translational regulatory elements [113]. Proteins of the translational machinery that are encoded by TOP mRNAs could be mentioned as ribosomal proteins [114], elongation factors eEF1A and eEF2 [115], and poly(A) binding proteins [116]. The expression and regulation of TOP mRNAs are not restricted to mammals, since they have been found in other vertebrates and even in drosophila and insects [117]. 5′ TOP of these mRNAs render them translationally suppressed upon cell cycle arrest caused by amino acid starvation, contact inhibition, and differentiation termination, which eventually make them sensitive to cellular stress signals and amino acid status [117, 118].

miR-10a was found to be highly expressed in kidney, muscle, lung, and liver of mice [119]. miR-10a was reported to interact with noncanonical downstream of the TOP motif in 5′UTR of ribosomal proteins and enhance their translation in a rapamycin-mTOR sensitive manner by alleviating their TOP-mediated translational repression during amino acid starvation [113, 120]. Consequently, miR-10a binding results in an elevation in global protein synthesis by means of enhancing the ribosomal protein yield and therefore affects the capability of cell transformation [120].

Recent miRNA profiling and mutational studies revealed that miR-346 is produced from the second intron of glutamate receptor ionotropic delta 1 (GRID1) mostly in brain tissues and is capable of upregulating RIP140 (receptor-interacting protein 140) gene via binding to 5′UTR of the target RIP140 mRNA and accelerating its target mRNA interaction with polysomes [121, 122]. RIP140 is a transcription coregulator and modulates many metabolism-related pathways by regulating nuclear receptors and transcription factors [123]. Nevertheless, miR-346 does not require AGO2 for its activity; therefore, it possibly applies an AGO-independent pathway to control the protein yield of RIP140 without altering its mRNA levels [122].

#### 4.1.2. miRNA-Mediated Upregulation in Competing with mRNA Decay and Expression Repressive Factors

miRNAs are able to compete with decay pathways such as AU-rich element- (ARE-) mediated decay pathway and other expression inhibitors. The ARE-mediated mRNA decay (AMD) regulates the concentration of a class of mRNAs that contains AU-rich sequences within their 3′UTRs. ARE-binding proteins (ABPs) recruit the cytoplasmic mRNA degradation machinery to the target mRNAs leading to their 3′-to-5′ degradation [124, 125]. Tristetraprolin (TTP) protein family functions as a molecular link between ARE-containing mRNAs and the mRNA decay machinery through degradation enzymes recruitment [126]. Noteworthy, it was indicated that some miRNA-mediated regulation pathways may have some interactions with ARE-mediated pathways, since they share common binding sites in mRNA 3′UTRs and have some common key players such as HuR, AGO2, CCR4, GW182, and decapping enzymes [127, 128]. Therefore, microRNAs have the capability to abrogate AMD by preventing ABPs associations leading to increased mRNA stability. miR-4661 and miR-125b are examples that hinder TTP binding to the ARE and, hence, increase IL-10 and *κ*B-Ras2 mRNA levels [129, 130].

Furthermore, some specific miRNAs have been recently found to block repressive proteins from binding to their target sites and, therefore, lead to distinct mRNA expression upregulation. miR-328 expression was found to be elevated in blast crisis chronic myelogenous leukemia (CML-BC) via the MAPK pathway, causing differentiation, impairing leukemic blasts survival through acting as a sponge molecule, decoying away hnRNP E2, a repressive protein, from C/EBP*α*, and eventually leading to C/EBP*α* expression upregulation [131].  Table 1 summarizes examples of miRNA-mediated upregulation.

### 4.2. Mechanisms of Derepression from miRNA-Mediated Downregulation

Derepression or relief of repression is the consequence of disengaging miRNPs from the previously repressed mRNAs [132]. This reversibility in miRNA-mediated gene regulation undoubtedly makes their functions more dynamic and brings the ability to be more receptive to specific cellular requirements.

#### 4.2.1. Relief of miRNA-Mediated Downregulation in Response to Cell Stresses

The Derepression of target mRNA in response to cell stress and synaptic stimulation is frequently mentioned examples [133, 134]. Cells recurrently run into stresses, including oxidative stress in cancer cells, especially in poorly angiogenic core of solid tumours [135, 136], nutrient deprivation [132, 137, 138], cardiac pressure overload [139], DNA damage, and oncogenic stress, resulting mostly from exposure to UV radiation [140–143] and salt imbalance [144].

As mentioned above, AMD and miRNAs have some interactions, and thereby miRNA could mediate or prevent AMD pathway. In this regard, the CAT-1 mRNA derepression would be a notable example which is accompanied by the relief from cytoplasmic bodies and its employment to polysomes. CAT-1 expression was found to be regulated comprehensively at both transcriptional and posttranslational levels and could be upregulated in response to different cellular stresses, such as amino acid deprivation in order to maintain hepatocellular protein synthesis [145]. Its upregulation requires HuR protein binding, an ARE-binding protein, to the 3′UTR of CAT-1 mRNA [146, 147]. HuR shuffles between nucleus and cytoplasm and was found to play a vital role in different posttranscriptional pathways not only in stress responses, but also in cell proliferation, differentiation, tumorigenesis, and immune responses [148, 149]. HuR binds to ARE sites of mRNAs in nucleus and chaperones them to the cytoplasm which then are relocalized in polysomes in response to stress. Moreover, it may also modulate translation or increase stability of target CAT-1 in some direct and indirect pathways [132].

**Figure 4:**
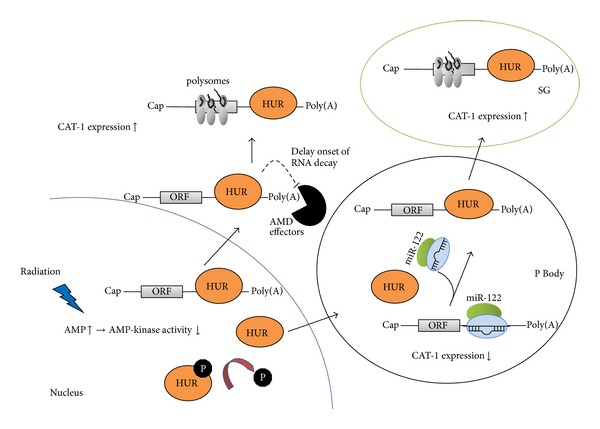
Relief of miRNA-mediated CAT-1 repression according to cell response to radiation. High AMP/ADP in cell is accompanied by the cell exposure to radiation. Consequently, AMP-kinase activity is enhanced, which then leads to HuR (an RNA binding protein that interacts with ARE in 3′UTR of the mRNA) dephosphorylation, releasing from nucleus to cytoplasm and entering to processing bodies (PBs). In P bodies, HuR dissociates miRNP from CAT-1 mRNA and mobilizes it to stress granules (SGs). Within SGs, CAT-1 expression is elevated via recruiting polysomes and relieved from miR-122-mediated downregulation by HuR replacement of miRNP in PBs. Moreover, HuR binds to ARE sites of mRNAs in nucleus and chaperones them to the cytoplasm which then are relocalized in polysomes and contributes in delays onset of RNA decay by competing with AMD effectors.

The clear mechanism that underlies HuR roles in posttranscriptional gene regulation remains poorly understood. However, the role of HuR in competing with other RNA binding proteins which function in promoting mRNA turnover is fairly confirmed [150, 151]. Although HuR does not have significant impact on poly(A) shortening, it contributes to delays onset of RNA decay [152]. Growing body of evidence supports that HuR has an RNA-stabilizing role in the ARE-directed mRNA decay in mammalian cells [153]. In addition to the the main points, HuR or similar regulatory proteins such as DND1 can influence the miRNA machinery interaction with target mRNA via dissociating miRNP from CAT-1 mRNA and relocating them into stress granules (SGs) which then results in polysomes recruitment [154] (Figure 4).

#### 4.2.2. Relief of miRNA-Mediated Downregulation by Sponge Molecules

Some sponge molecules such as lncRNAs and AGO10 were reported to decoy away miRNAs from their target mRNAs and lead to target mRNA derepression. One type of these lncRNAs is the so-called “miRNA sponges.” They bind to specific miRNAs in their seed site and prevent miRNP binding to their target mRNAs, or they compete with miRNAs for binding to the specific mRNAs [155, 156]. BACE1-AS is one of these examples located in the antisense strand of BACE1 (beta-secretase 1) and competes with miR-485-5p for binding to the exon 6 of BACE1 mRNA [157]. Hence, BACE1-AS expression is associated with BACE1 mRNA stability and increases the protein yield of BACE1 [158]. Linc-ROR is another miRNA sponge expressed in the pluripotent stem cells and increases reprogramming efficiency [159–161]. Core pluripotency transcription factors like OCT4, SOX2, and NANOG mRNAs are the miR-145 target RNAs. However, in the presence of Linc-ROR, miR-145 is trapped and consequently maintains the self-renewal state of stem cell due to the increasing stability of these three transcription factor's mRNAs and subsequently the increasing level of their proteins [162]. These transcription factors result in embryonic stem cells-specific gene expression, which prevents stem cell differentiation [163].

In* Arabidopsis*, a decoy Argonaute protein, called AGO10, recruits miR-166/165 by recognizing its distinctive secondary structure and decoys it away from AGO1 and consequently leads to target mRNA, homeodomain leucine zipper transcription factors, and expression upregulation, which maintains undifferentiated cells of the shoot apical meristem [164].  Table 2 summarizes some examples of derepression of miRNA-mediated downregulation.

## 5. Concluding Remarks and Future Challenges

Accumulating reports had brought about the estimation that over 3 percent of human genes in human genome are subjected to miRNA-mediated gene regulation in different cell processes, suggesting that the expression of this important noncoding RNAs are associated with an array of pathological outcomes and human disease. MicroRNAs have several characteristics that make them an intriguing candidate for cell protection. As advances in the field of miRNA-mediated gene regulation are made, it is apparent that miRNAs are a crucial component of gene regulatory networks. While, most studies dedicated a downregulation role for miRNA-mediated post transcriptional gene regulation, recently increasing publications reveal an adverse role for miRNAs as activators of gene expression. miRNPs enhance protein yield of target mRNA by mRNA degradation and/or translational repression. Nevertheless, miRNA-mediated upregulation of target mRNA can be elucidated by both enhancing mRNA stability and translational activation via direct activation and/or indirect derepression. Despite the rapid progress and a wealth of information about miRNP-mediated upregulation, the general molecular mechanism of switching from repression to activation has only been delineated in a few distinct conditions and tissues.

However, the aforementioned cell responses resulting in gene expression upregulation could not be generalized to all miRNAs or tissues. For example, miR-34a targets AXIN2 through binding to its 5′UTR and downregulates its expression. Also, several miRNAs have been found to suppress gene expression even in G0 state cells and the cells which endure any type of stresses [58]. Perhaps the most puzzling and interesting aspect of posttranscriptional gene regulation (PTG) by miRNA is that PTG is not carried out only by miRNAs, since numerous well-documented examples of PTG mediated by molecules and processes other than miRNAs are present. Surprisingly, the cooperation between the cellular environment, mRNA context, interplay between other PTGs, and miRNA-mediated gene regulation dictates the fate of the target mRNA. Hence, PTG is involved in several distinct and most likely overlapping mechanisms. Given this complexity, it will be important to define which mechanism is exerted for regulation of a special subset of mRNAs. Nonetheless, classifying mRNAs in accordance with their regulational subtypes would be difficult as one PTG mechanism is capable of mediating several gene expressions in the meantime, and vice versa a single mRNA can be a subject of different PTG processes. More challenging will be the identification and characterization of cell type and condition in order to define a unique mechanism for a unique gene in response to each different factor.

In conclusion, this exiting playground of miRNA-mediated gene regulation still holds secrets, and discrepancies in their studies invite future cellular, molecular, and biochemical studies, as well as computational approaches, to uncover their molecular mechanism, in order to provide a new dimension to the understanding, prevention, and treatment of human diseases.

## Figures and Tables

**Table 1:** Examples of direct miRNA-mediated upregulation.

miRNA	Target mRNA	Expression regulation	References
miR-360-3p	TNF*α*	miRNA recruits a modified microRNP with AGO2 and FXR1-iso-a and mediates translation activation.	[82, 85, 86]
miR-206	KLF4	In G0 state cells and nontransformed cells, the interaction of GW182 and AGO2 is restricted and FXR1a alters the function of miRNP.	[85]
xlmiR-16	Myt1	In immature *Xenopus laevis* oocytes dAGO inhibits the interaction of GW182 with miRNP which leads to a loss of repression.	[100]
miR-122	HCV	miRNA directly binds to two target sites in 5′UTR of HCV RNA and increases its association with 40S and polysomes formation.	[107, 108]
miR-10a	TOP RNA	miR-10a interacts with 5′UTR of ribosomal proteins and enhances their translation by alleviating their TOP-mediated translational repression during amino acid starvation.	[120]
miR-346	RIP140	A target sequence for miRNA miR-346 was found in the 5′UTR of RIP140 mRNA. miR-346 elevates RIP140 protein levels by facilitating association of its mRNA with the polysomes fraction.	[122]
miR-34a/b-5	*β*-Actin	Beta-actin (Actb) gene generates two alternative transcripts terminated at tandem poly(A) sites. The longer transcript harbours a conserved mmu-miR-34a/34b-5p target site. miR-34 binding to Actb 3′-UTR upregulates target gene expression.	[165]
miR-125b	B-Ras2	miRNA prevents TTP binding to the ARE sites of B-Ras2 and inhibits its degradation by AMD pathway.	[130]
miR-328	C/EBP*α*	miRNA decoys away hnRNP E2, a repressive protein, and upregulates its expression in a seed sequence independent manner.	[131]

**Table 2:** Examples of relief from miRNA-mediated downregulation.

miRNA	Target mRNA	Expression regulation	Reference
miR-122	CAT-1	In response to amino acid starvation, HuR binding to 3′UTR interferes with miRNP association with CAT-1 mRNA and results in relocalization of mRNP from P bodies in the cytoplasm to polysomes.	[105, 110]
miR-134	LIMK1	In response to extracellular stimuli, HuR binds to LIMK1 mRNA in the dendritic spines and alleviates miRNA-mediated repression.	[166, 167]
miR-19	RhoB	The loss of the interdependent binding between HuR and miR-19 to the RhoB mRNA upon UV exposure relieves this mRNA from miR-19-dependent inhibition of translation.	[168]
miR-548c-3p	TOP2A	HuR enhances TOP2A translation by antagonizing with miR-548c-3p. Their combined actions control TOP2A expression levels and determine the effectiveness of doxorubicin.	[169]
miR-430	Nanos1 and TDRD7	DND1 prevents miRNA-mediated downregulation in primordial germ cells by blocking miRNP access to 3′UTR of target mRNAs.	[154]
miR-221 and miR-222	P27KIP1	In arrested cells, Pum1 is unable to bind to 3′UTR of target mRNA and cannot open its loop structure and therefore restricts miRNA binding.	[154]
miR-19b	PTEN	PTEN1 (a pseudogene) acts as a decoy factor for miRNAs and derepresses PTENs expression.	[170]
miR-20a	KRAS	KRAS1P sequesters miRNA and promotes expression of KRAS.	[170]
miR-166/165	Homeodomain leucine zipper transcript factor	AGO10 acts as a sponge to decoy away miRNAs from AGO1 bearing miRNPs.	[164]
miR-184 and let-7	LRRK2	Gain of function mutation in LRRK2 makes it more associable to dAGO1 and LRRK2 kinase which phosphorylates 4E-BP1 and consequently associates with hAGO2 which counteracts with miRNA mediated repression.	[171, 172]
miR-26a/b	IL-6	3′-end uridylation of miRNAs relieves their miRNA-mediated repression and promotes IL-6 expression.	[173]
miR-485-5p	BACE1	BACE1-AS (a lncRNA) binds to BACE1 mRNA in its seed site and prevents miRNP binding to their target mRNAs.	[158]
miR-145	OCT4, SOX2, and NANOG	In the presence of Linc-ROR (a lncRNA), miR-145 is trapped and consequently decoyed away from its target mRNAs.	[159–162]
